# Effects of implementing permissive campus carry laws on rates of major violence at public colleges and universities

**DOI:** 10.1186/s40621-025-00566-0

**Published:** 2025-03-03

**Authors:** Rose M. C. Kagawa, Paul M. Reeping, Hannah S. Laqueur

**Affiliations:** Centers for Violence Prevention, Department of Emergency Medicine, University of California at Davis, 4301 X St., Sacramento, CA 95817 USA

**Keywords:** Firearm injury, Firearm policy, Campus carry, Concealed carry

## Abstract

**Background:**

Following the Supreme Court’s decision in New York State Rifle & Pistol Association, Inc. v. Bruen, which ruled a New York concealed-carry permitting requirement unconstitutional, laws restricting the public carrying of firearms in “sensitive places,” like college campuses, have received increasing attention. However, there is little evidence for whether permissive campus carry policies increase firearm violence or, via deterrence, reduce general crime on campus. We estimated the effect of implementing state laws allowing the carry of firearms on public college and university campuses on rates of violent crime and burglary.

**Methods:**

Arkansas, Georgia, and Texas, containing 106 public institutions, implemented permissive campus carry laws in 2017, 2017, and 2016, respectively. Control institutions were all those in states that did not allow the carry of firearms on college campuses for the entire study period (2006–2019) (n = 324 institutions, 21 states). The rates of major violence and burglary per 1,000 enrolled students was obtained from the Office of Postsecondary Education Campus Safety and Security Statistics website. We use two-way fixed effects difference-in-differences models to estimate state-specific effects and a modified difference-in-differences approach that accounts for variation in treatment timing to generate an overall estimate.

**Results:**

Differences in rates of major violence and burglary were not statistically distinguishable from zero in our main models and sensitivity analyses. The overall estimated difference in the rate of major violence following policy implementation was − 0.01 (− 0.113, 0.093). For burglary, we estimated a difference of − 0.02 (− 0.147, 0.106). Violence rates trended upward in treated states in the last exposure period, but differences were not consistently distinguished from the null.

**Conclusions:**

This study does not find significant changes in crime rates following state implementation of permissive campus carrying laws. Decision-makers might therefore consider other factors such as the opinions of students, faculty, and staff regarding campus carry policies and feelings of safety, potential impacts on instructional quality and student engagement, and potential impacts on accidental or self-directed harm.

**Supplementary Information:**

The online version contains supplementary material available at 10.1186/s40621-025-00566-0.

## Introduction

Location-restriction laws or so-called “Gun Free Zones,” represent one approach to reducing firearm injuries and deaths. These laws aim to reduce the risk and lethality of conflicts arising in “sensitive places” that host vulnerable populations, or inpotentially high-risk places or situations. For instance, many states prohibit firearms in venues where large crowds are present, such as sports stadiums or during demonstrations and protests, as well as in places where activities could heighten the likelihood of violence, such as establishments serving alcohol [1]. These laws have garnered more attention following the Supreme Court’s decision in New York State Rifle & Pistol Association v. Bruen, which ruled specific aspects of New York’s concealed-carry permitting system unconstitutional [2].

Although location-restriction laws are intended to reduce firearm violence, some argue that disallowing citizens from carrying firearms leaves these locations and the people in them undefended and thus could increase the risk of victimization. High-profile mass shootings at schools and colleges frequently reignite debates regarding firearms in educational settings and have prompted legislation governing their carrying in these institutions [3]. For example, in the aftermath of the 2012 Sandy Hook elementary school shooting, lawmakers in more than 30 states proposed bills to authorize schools to arm and train teachers and staff to serve as armed security. Following the high-profile Virginia Tech mass shooting in 2007 in which 32 people were killed, there was renewed debate regarding public firearm carrying laws and regulations on college campuses [4]. Since then, several states have passed legislation allowing the concealed carry of firearms on college campuses, with proponents suggesting that this allows students and faculty to better defend themselves in an attack. Other states have outright prohibited firearms on college campuses or adopted a mixed approach that either confines firearms to specific areas on campus or grants colleges the authority to formulate their own firearms policies. Some states have also passed laws that expressly prohibit college and university campuses from designating themselves gun free, although still often allowing prohibitions in certain areas [5].

Policies restricting firearms on college and university campuses are generally viewed positively by students and faculty. A systematic review of seventeen studies examining attitudes toward campus carry by students, staff, and faculty, found that, across all studies, the majority either did not agree firearms should be allowed to be carried on campus and/or believed that carrying of firearms on campus makes them less safe [6]. Other surveys have found that professors are also worried that the presence of firearms would limit their right to free speech [7], or would impact the way they communicate with students [8].

Despite the enactment of campus carry laws across the country, there is limited empirical evidence on the impacts of these laws. Studies using a small sample of states and study years have generally found no significant association between campus carry laws and violent crime [9, 10]. To date, there has been only one study using more recent data and a more complete sample of states over time to test whether allowing students and faculty to carry firearms on campus has a deterrent effect [11]. It did not find that campus carry laws were associated with an increase in violence or property crime. However, the study focused on within state change rather than comparing the change in treated states to the change in a set of control states. Further, it examined law changes in ten states, even though in many of these states, concealed carrying was prohibited in practice due to laws permitting campuses to regulate firearms. Our study adds to the existing evidence by using a robust difference-in-differences estimation approach and defining treated states more stringently so as to examine starker changes in permissive campus carry policies relative to a control group of states that prohibited concealed carry.

In this study, we leverage temporal variation in the passage of campus carry laws across states to test whether the passage of permissive campus carry laws is associated with lower rates of violent crime and burglary on public college campuses. Specifically, we evaluate the impact of Arkansas, Georgia, and Texas’ implementation of permissive campus carry laws in 2017, 2017, and 2016, respectively compared to control states that did not allow the carry of firearms on college campuses for the entire study period (2006–2019). We focus on only those states that explicitly passed statutes allowing campus carry and where, in practice, colleges and universities allowed carrying in most campus locations.

## Methods

The study population includes all public institutions with onsite student housing in the 24 states with policy environments that met eligibility criteria, described below. Private institutions were excluded as these have greater ability to establish their own gun carrying policies. No limitations were placed on enrollment or instructional type.

The study period begins in 2006, 10 years prior to the first policy change, and ends in 2019. We exclude the years 2020 and 2021 because we do not have information on COVID-19 related stay-at-home orders or practices across institutions.

States that changed their campus carry policy from explicitly prohibited to explicitly permitted were considered “treated.” To make this determination, we reviewed state campus carry laws as well as more general concealed carry laws (shall issue, may issue, permitless) summarized in RAND Corporation’s State Firearm Law Database; Armedcampuses.org, produced by The Campaign to Keep Guns Off Campus and The Coalition to Stop Gun Violence; and previous publications on this topic [12, 13]. Our treated states include those that moved from explicitly prohibiting to explicitly allowing the carry of firearms on public university and college campuses with few exceptions (e.g. allowance for law enforcement officers and in cars).

During our study period, Arkansas, Georgia, and Texas, representing 106 public institutions, implemented permissive campus carry laws in 2017, 2017, and 2016 respectively. All three states enacted laws exempting concealed carry permit holders from preexisting campus carry restrictions. In Texas, the exemption first applied to all 4-year institutions and was expanded to 2-year and junior colleges in 2017. No other states met our criteria for treatment. Treatment timing is based on the date of implementation. If a state implemented the policy of interest in the first six months of a year, this year was included in the post-treatment period, and otherwise in the pretreatment period.

Control states include all those that had a state law prohibiting the carry of firearms on campus or in which there was no law and all institutions prohibited the carry of firearms on campus grounds during the entire study period (324 institutions, 21 states) based on Armedcampuses.org’s assessment.

We are unable to estimate the effects of states implementing restrictive campus carry policies. States that enacted laws moving from a permissive to a more restrictive campus carry policy environment did so in the early 2000s, leaving too little pre-treatment time to allow for estimating effects with the current data. New York implemented a restrictive campus carry policy in 2013, but as a May-Issue state, this change was less substantial than our eligibility criteria allowed.

We estimate effects for two outcomes: major violent crime per 1,000 enrolled students and burglary per 1,000 enrolled students. Major violent crime includes murder, rape (“forcible sex” prior to 2014), robbery and aggravated assault. The outcome data are from the Office of Postsecondary Education Campus Safety and Security (CSS) Statistics website. Reporting to this database is required for all institutions that receive Title IV funding. In terms of property crimes, the CSS reports data on arson, burglary, and motor vehicle theft. We hypothesized burglaries would be most plausibly influenced by changes to campus carry policies.

CSS reports enrollment at the institution level and crimes at the campus level. One institution had an out-of-state campus. Because firearm carry policies differ across states, crimes occurring at the Preservation Institute of Nantucket were removed from the University of Florida. We also removed institutions that did not report data for the full study period (n = 22).

Due to the staggered timing of policy implementation, we used a modified difference-in-differences approach, which accounts for variation in treatment timing (Callaway and Sant'Anna, 2021). We present average effect estimates by time since exposure along with 95% confidence intervals calculated using bootstrap-based standard errors and adjusted for multiple testing. We additionally apply standard two-way fixed effects difference-in-differences models to estimate effects for each state individually.

We conducted several sensitivity analyses. First, we tested whether results are sensitive to the inclusion of covariates that we hypothesized might confound the relationship of interest. Covariates included the percentage of the state population that voted for the Republican presidential candidate in the elections 2004–2016, sourced from the American Presidency Project at the University of California, Santa Barbara [14], and the state firearm homicide rate from the Centers for Disease Control and Prevention. Both variables may be associated with the chances of passing state-wide campus carry laws, as well as the prevalence of firearm carrying. Institution-level variables include the proportion of enrolled students who are male, the proportion receiving Pell grants, and the proportion racialized as Black or African American. These variables were sourced from the National Center for Education Statistics [15], and represent populations at higher risk of being exposed to or involved in violence [16].

We also tested whether the results differed when using institutions as the unit of analysis, excluding from the control group states that had no specific state-level policy, and excluding rape counts from the major violent crime outcome. The last was tested because rape counts at institutions sometimes far outnumbered all other crime types considered.

Finally, we explored whether the results were robust to alternative modeling approaches. First, we used Augmented Synthetic Control with Staggered Adoption. Traditional difference-in-differences methods can fail when the parallel trends assumption is not met. Synthetic control with staggered adoption uses a weighted difference-in-differences that can accommodate non-parallel pre-treatment trends more flexibly than standard difference-in-differences methods and optimizes both overall and state-specific fit. Because we tested only three policy changes occurring over a short period of time (2016 and 2017), we estimated effects using a traditional two-way fixed effects model. Our third approach employed Wooldridge’s Mundlak regression, which relaxes the common trends assumption and adjusts for heterogenous treatment effects across time or treatment group when treatment timing is staggered [17].

## Results

**Fig. 1:**
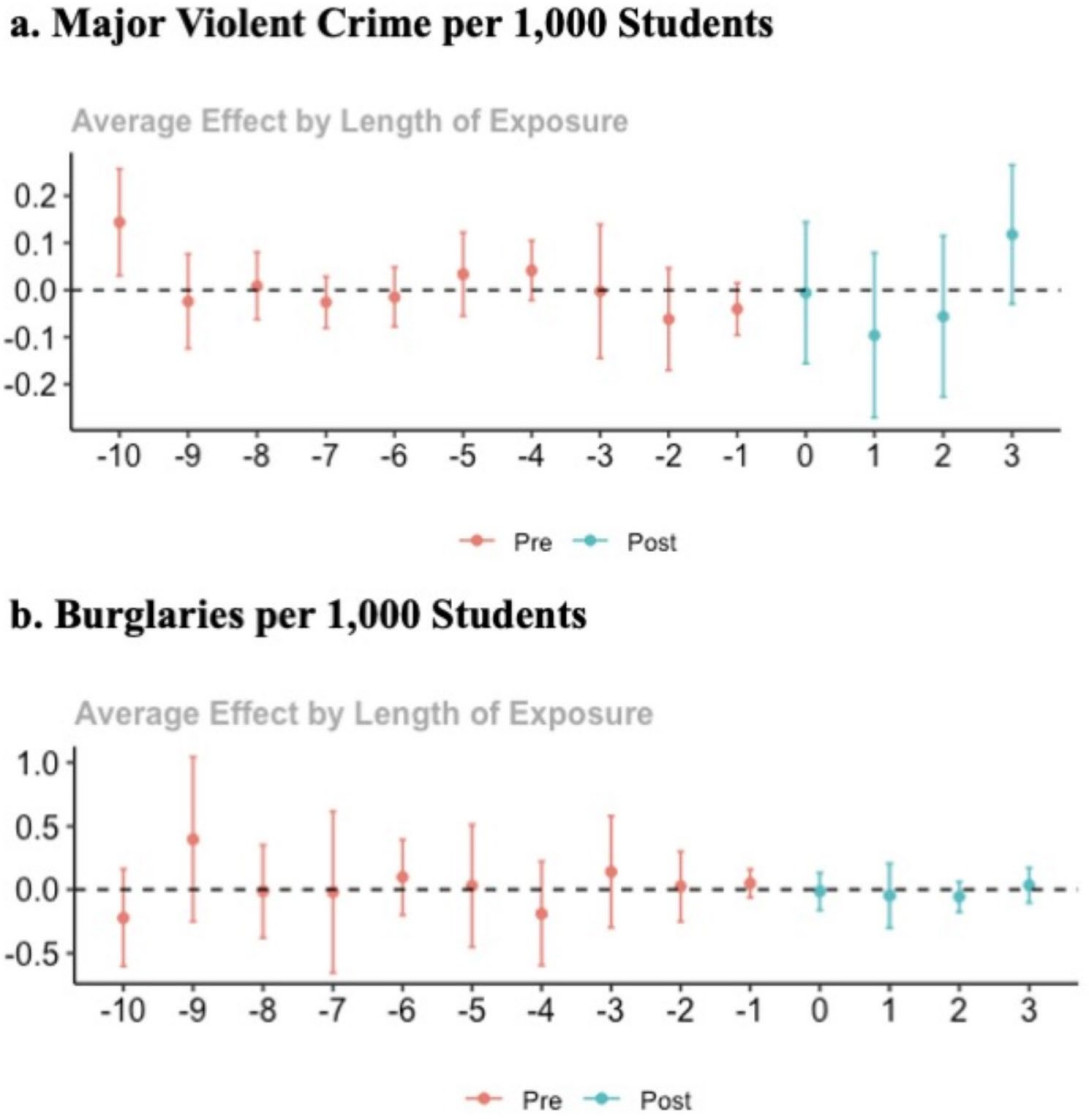
Differences between treated and control states by year relative to treatment time (0)

We assessed the parallel trends assumption visually (Fig. 1) and using statistical tests for pre-treatment differences. There is minimal evidence of deviation from parallel trends in the pre-intervention period with only the first time period (10 years pre-treatment) showing a statistically significant difference in the rate of violent crime between control and not yet treated states (0.14, 95% CI = 0.026–0.261). This difference is also apparent in Fig. 1.

Differences in the post-treatment period are small and not well distinguished from the null, with the largest post-period difference suggesting an increase of 0.12 (− 0.030, 0.265) major violent crimes per 1,000 students three years after policy change. The average rate of violent crimes per year in the sample is 0.58, with a standard deviation of 0.32. The overall estimated average treatment effect was − 0.01 (− 0.113, 0.093).

Similar results are observed for the burglary rate, with the largest difference suggesting a decrease of 0.06 (− 0.169, 0.057) burglaries per 1,000 students in year two and an estimated overall average treatment effect of − 0.02 (− 0.147, 0.106). The average burglary rate over the study period is 1.27 per 1,000 students (SD = 0.89).

Results from the state specific models are shown in Table 1. None of the differences estimated from these state-specific difference-in-differences models were statistically distinguishable from zero.

**Table 1:** Average treatment on the treated estimates by outcome and state

	Difference	95% CI
*Violent crime rate*		
Arkansas	− 0.15	− 0.423, 0.132
Georgia	− 0.11	− 0.389, 0.169
Texas	− 0.04	− 0.286, 0.212
*Burglary rate*		
Arkansas	− 0.25	− 1.164, 0.668
Georgia	0.13	− 0.774, 1.040
Texas	0.43	− 0.350, 1.204

When using institutions as the unit of analysis, differences are more precisely estimated and remain null. All other sensitivity analyses produce similar results to those shown here in that they are not statistically distinguishable from zero. However, in some cases the observed association is in the opposite direction. Additionally, when we control for state-level covariates, there is evidence of increasing rates of violence in the last observed year, which is three years following policy change (ATT = 0.04; 95% CI = 0.008, 0.187). Results from the differing statistical approaches can be found in the appendix.

## Discussion

Our results do not provide clear evidence for a decrease or increase in the rates of major violent crime or burglary on public college and university campuses following the implementation of more permissive campus carry policies. There have been few previous studies examining this question, though those that have reached similar conclusions [9–11, 13]. The current study is unique in that it uses a difference-in-differences framework to estimate effects and defines treated and control units to examine starker changes in campus carry policy (e.g., from prohibited to allowed rather than from no explicit law in a shall issue state to allowed).

Even with these considerations, the policy changes considered are nuanced and likely constitute weak treatments [18]. For example, firearm carrying in all three states considered is only allowed for those 21 and over, unless the individual is in the Armed Forces. As a result, the policy impacts only a subset of the enrolled student population. In Arkansas, individuals can only carry on campus after they have obtained an enhanced permit, which requires additional training beyond that which is required for a concealed carry permit. Additionally, many institutions limit the carrying of firearms in specific places on campus. Sports arenas, anywhere minors are present, and even residence halls are sometimes excluded.

We are unable to estimate effects on rates of firearm violence specifically, which would provide a better test of the hypothesis that increased access to firearms on college and university campuses increases firearm violence rates. We also do not have information on actual firearm carrying at the university level and, were we to interpret the present result as no effect, we still cannot know whether the observed results stem from little change in access to firearms or little change in the downstream effects of increased firearm carrying (either protective or harmful). Because of data availability and movement toward more permissive firearm carrying policies in the US, we were not able to study the impact of moving to a more restrictive carrying environment. Institution-specific policies, which influence the selection of states for our control group, were defined by armedcampuses.org and we could not ascertain change over time. However, we conducted a sensitivity analysis excluding states that allowed individual institutions to determine their campus carry policy, finding no meaningful difference in our results. Finally, our sample includes only three treated states, limiting both statistical power and generalizability.

Null results on their own do not provide clear guidance for decision-makers, but there are other factors that are important to consider. For example, given the lack of evidence relating to effects on interpersonal violence and property crime, campus administrators and policymakers might more heavily weigh the opinions of students, faculty, and staff regarding campus carry policies and feelings of safety. Overwhelmingly, these studies show campus community members tend to feel less safe when firearms are allowed [6, 19–23]. These studies also tend to note variation in attitudes, depending on familiarity with firearms, fear of victimization, minoritized status, regional origin, or political leanings. Additionally, there may be impacts on instructional quality and student engagement. Research from Texas indicates faculty have changed how they teach following the implementation of campus carry, for example limiting office hours or holding them in public places and avoiding the discussion of sensitive topics in class [24]. Similarly, faculty in Georgia reported avoiding controversial topics in class discussions, the pursuit of academic misconduct charges, and other activities that could be perceived as unsafe [25]. However, 51% of the respondents did not report experiencing any of the negative impacts of having guns on campus that were asked about.

Finally, there may be effects on accidental or self-directed harm, which were not considered in the current study. College campuses are home to large populations of emerging adults, which warrants special consideration with regard to risky behavior and self-harm [12]. Some risky behaviors, like binge drinking, peak at this age, increasing risk for violent altercations. This is also a vulnerable age when it comes to mental health and mental illness, and increasing access to firearms is associated with increased rates of suicide [26]. In the absence of a clear effect on interpersonal violence, decisionmakers are encouraged to consider the evidence on these related factors.

## Supplementary Information

Additional file1

## Data Availability

The datasets used and analyzed during the current study are available from the corresponding author on reasonable request. Data used in the current study are available for download online: Outcome data: U.S. Department of Education. Campus Safety and Security. Download Custom Data. 2024. https://ope.ed.gov/campussafety/#/ Policy data: Cherney, Samantha, Andrew R. Morral, Terry L. Schell, Sierra Smucker, and Emily Hoch, Development of the RAND State Firearm Law Database and Supporting Materials. Santa Monica, CA: RAND Corporation, 2022. https://www.rand.org/pubs/tools/TLA243-2-v2.html, and Armed Campuses, A project of The Campaign to Keep Guns Off Campus and The Coalition to Stop Gun Violence. Certain content provided by The Law Center to Prevent Gun Violence. 2016. https://www.armedcampuses.org.
