# Supplementary material for: Effects of implementing permissive campus carry laws on rates of major violence at public colleges and universities
Source: Inj Epidemiol. 2025 Mar 3;12:14. doi: 10.1186/s40621-025-00566-0 (PMC11874404; doi:10.1186/s40621-025-00566-0)
Supplement: Supplementary file 1 — Additional file1 [file 40621_2025_566_MOESM1_ESM.docx]

Appendix to “Effects of Implementing Permissive Campus Carry Laws on Rates of Major Violence at Public Colleges and Universities”

Authors: Rose Kagawa, Paul Reeping, Hannah Laqueur

**Appendix A. Results from Augmented Synthetic Control with Staggered Adoption Models**

Average ATT Estimate for Violent Crime Rate= -0.080 (-0.365, 0.187)

Average ATT Estimate for Burglary Rate= 0.015 (-0.415, 0.489)

Appendix Figure 1. Differences in Violent Crime Rates Between Each Treated State and Its Synthetic Control Plotted Over Time Relative to Treatment


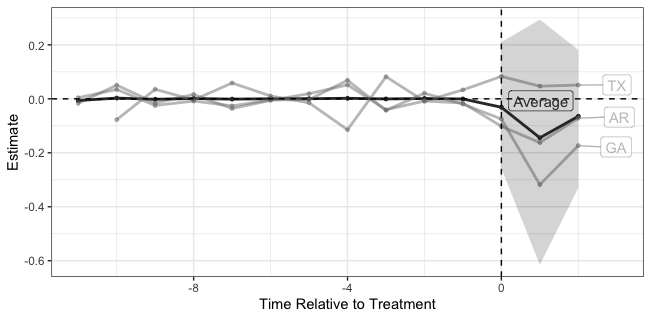


Appendix Figure 2. Differences in Burglary Rates Between Each Treated State and Its Synthetic Control Plotted Over Time Relative to Treatment


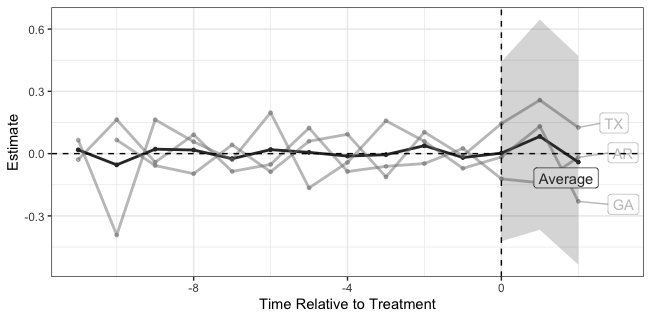


**Appendix B. Results from Traditional, Two-Way Fixed Effects Models**

| **Outcome** | **DID Estimate** | **95% Confidence Interval** |
| --- | --- | --- |
| Violent Crime Rate  (per 1,000) | -0.09 | -0.241, 0.065 |
| Burglary Rate  (per 1,000) | 0.16 | -0.328, 0.653 |
| Violent Crime Rate (excluding rape)  (per 1,000) | 0.02 | -0.053, 0.092 |

**Appendix C. Results from Wooldridge’s Two-Way Mundlak Regression**

| **Outcome** | **DID Estimate** | **95% Confidence Interval** |
| --- | --- | --- |
| Violent Crime Rate  (per 1,000) | -0.09 | -0.194, 0.012 |
| Burglary Rate  (per 1,000) | 0.14 | -0.146, 0.419 |
| Violent Crime Rate (excluding rape)  (per 1,000) | 0.02 | -0.023, 0.058 |

References:

Fernando Rios-Avila & Arne J. Nagengast & Yoto V. Yotov. 2022. "JWDID: Stata module to estimate Difference-in-Difference models using Mundlak approach," Statistical Software Components S459114, Boston College Department of Economics, revised 08 Apr 2024.

Wooldridge, Jeffrey M. 2021. “Two-Way Fixed Effects, the Two-Way Mundlak Regression, and Difference-in-Differences Estimators,” SSRN Scholarly Paper. Rochester, NY. https://doi.org/10.2139/ssrn.3906345.
